# The Origin of Tumor DNA in Urine of Urogenital Cancer Patients: Local Shedding and Transrenal Excretion

**DOI:** 10.3390/cancers13030535

**Published:** 2021-01-31

**Authors:** Anouk E. Hentschel, Rianne van den Helder, Nienke E. van Trommel, Annina P. van Splunter, Robert A. A. van Boerdonk, Mignon D. J. M. van Gent, Jakko A. Nieuwenhuijzen, Renske D. M. Steenbergen

**Affiliations:** 1 Amsterdam UMC, Vrije Universiteit Amsterdam, Pathology, Cancer Center Amsterdam, de Boelelaan 1118, 1182 DB Amsterdam, The Netherlands; a.hentschel@amsterdamumc.nl (A.E.H.); r.vandenhelder@amsterdamumc.nl (R.v.d.H.); a.vansplunter@amsterdamumc.nl (A.P.v.S.); r.boerdonk@amsterdamumc.nl (R.A.A.v.B.); 2 Amsterdam UMC, Vrije Universiteit Amsterdam, Urology, Cancer Center Amsterdam, de Boelelaan 1118, 1182 DB Amsterdam, The Netherlands; j.nieuwenhuijzen@amsterdamumc.nl; 3 Antoni van Leeuwenhoek/Netherlands Cancer Institute, Gynecologic Oncology, Center of Gynecologic Oncology Amsterdam, Plesmanlaan 121, 1066 CX Amsterdam, The Netherlands; n.v.trommel@nki.nl; 4 Amsterdam UMC, Location Amsterdam Medical Center, Gynecologic Oncology, Center of Gynecologic Oncology Amsterdam, Meibergdreef 9, 1105 AZ Amsterdam, The Netherlands; m.d.vangent@amsterdamumc.nl

**Keywords:** urine, biomarkers, liquid biopsy, methylation, mutation, molecular diagnostics, urogenital neoplasms

## Abstract

**Simple Summary:**

There is a clinical need for non-invasive methods to detect urogenital cancer, for which urine offers a promising liquid biopsy. Tumor DNA in urine originates from shedding of the local tumor, but may also be excreted transrenally as circulating DNA fragments from the bloodstream. To assess the origin of tumor-associated DNA in the urine of urogenital cancer patients, molecular tumor markers were tested in natural voided urine, and in urine samples in which contact with the local tumor was circumvented. The latter concerned nephrostomy urine of bladder cancer patients and catheter urine of cervical cancer patients. We demonstrated that tumor DNA in the urine of urogenital cancer patients results not only from the shedding of the local tumor, but also from transrenal excretion of circulating tumor DNA. This supports the great potential of urine as a diagnostic tool for cancer detection.

**Abstract:**

In urogenital cancers, urine as a liquid biopsy for non-invasive cancer detection holds great promise for future clinical application. Their anatomical position allows for the local shedding of tumor DNA, but recent data indicate that tumor DNA in urine might also result from transrenal excretion. This study aims to assess the origin of tumor-associated DNA in the urine of 5 bladder and 25 cervical cancer patients. Besides natural voided urine, paired urine samples were collected in which contact with the local tumor was circumvented to bypass local shedding. The latter concerned nephrostomy urine in bladder cancer patients, and catheter urine in cervical cancer patients. Methylation levels of *GHSR*, *SST,* and *ZIC1* were determined using paired bladder tumor tissues and cervical scrapes as a reference. Urinary methylation levels were compared to natural voided urine of matched controls. To support methylation results, mutation analysis was performed in urine and tissue samples of bladder cancer patients. Increased methylation levels were not only found in natural voided urine from bladder and cervical cancer patients, but also in the corresponding nephrostomy and catheter urine. DNA mutations detected in bladder tumor tissues were also detectable in all paired natural voided urine as well as in a subset of nephrostomy urine. These results provide the first evidence that the suitability of urine as a liquid biopsy for urogenital cancers relies both on the local shedding of tumor cells and cell fragments, as well as the transrenal excretion of tumor DNA into the urine.

## 1. Introduction

Urine as a liquid biopsy for non-invasive cancer diagnosis is gaining interest in different cancer types [1]. As expected from a biological perspective, urogenital cancers have proven to be particularly suitable for non-invasive cancer detection in urine [2,3,4]. Their anatomical position allows for local shedding of tumor cells and cell fragments into the urine, either directly (e.g., bladder cancer) or via vaginal excretion (e.g., cervical cancer) [1,5]. We recently demonstrated bladder and cervical cancer detection by DNA methylation analysis in different urine fractions obtained by centrifugation [6,7]. Both cancer types were detectable in urine sediment, mainly containing cellular DNA, as well as urine supernatant, which is supposedly enriched for cell-free DNA. Hence, the origin of tumor-associated DNA in the urine of urogenital patients is suggested to be attributed to the local shedding of tumor cells and cell fragments, but might also result from the transrenal excretion of tumor DNA [8,9].

The presence of cell-free DNA in urine by the transrenal excretion of tumor DNA is supported by the detection of tumor DNA fragments in the urine of non-urogenital cancer types such as lung, colorectal, and hepatocellular cancer [10,11,12]. It is hypothesized that tumor-associated DNA is released into the bloodstream and reaches the urinary tract via glomerular filtration as circulating cell-free tumor DNA [1]. However, no formal evidence for the latter route exists for urogenital cancers. Therefore, this study aims to provide further proof of the transrenal excretion of circulating tumor DNA in the urine of patients with urogenital cancers.

For this purpose, we collected paired bladder tumor tissues, natural voided urine, and urine derived from a kidney nephrostomy in five muscle-invasive bladder cancer patients. Additionally, paired cervical scrapes, natural voided urine, and catheter urine derived from the bladder were collected from 25 cervical cancer patients. In these cases, local shedding was bypassed by collecting nephrostomy urine in bladder cancer patients, and catheter urine in cervical cancer patients. Samples were tested for DNA methylation and DNA point mutations, representing extensively studied urinary biomarkers with high diagnostic potential for urogenital cancer detection [3,13,14]. DNA hypermethylation in promoter regions of tumor-suppressor genes can induce gene silencing, thereby contributing to cancer development [15]. We tested for DNA hypermethylation in promoter regions of *GHSR*, *SST,* and *ZIC1*, which enables the accurate detection of both bladder and cervical cancer in urine [4,6,16,17]. To verify methylation results in urine, corresponding bladder tumor tissues and cervical scrapes, which are enriched with local tumor DNA, were included as a reference [16,18]. In bladder cancer patients, DNA point mutations in hotspot regions of 37 cancer-related genes were studied to support the methylation results. These include 7 genes that are known to be associated with bladder cancer (*FGFR3*, *HRAS*, *KRAS*, *NRAS*, *PIK3CA*, *TERT* promoter, and *TP53*) [19,20]. DNA point mutations in *FGFR3*, the *RAS* genes, and *PIK3CA* are primarily linked to non-muscle-invasive bladder cancer, whereas *TP53* mutations are mainly detected in muscle-invasive bladder cancer, and mutations in the *TERT* promoter region are found across all bladder cancer grades and stages [19,21].

We demonstrate that the suitability of urine for urogenital cancer detection with molecular biomarkers is based on both local shedding and the transrenal excretion of tumor DNA into the urine.

## 2. Results

### 2.1. Methylation Markers in Bladder Cancer

**Figure 1:**
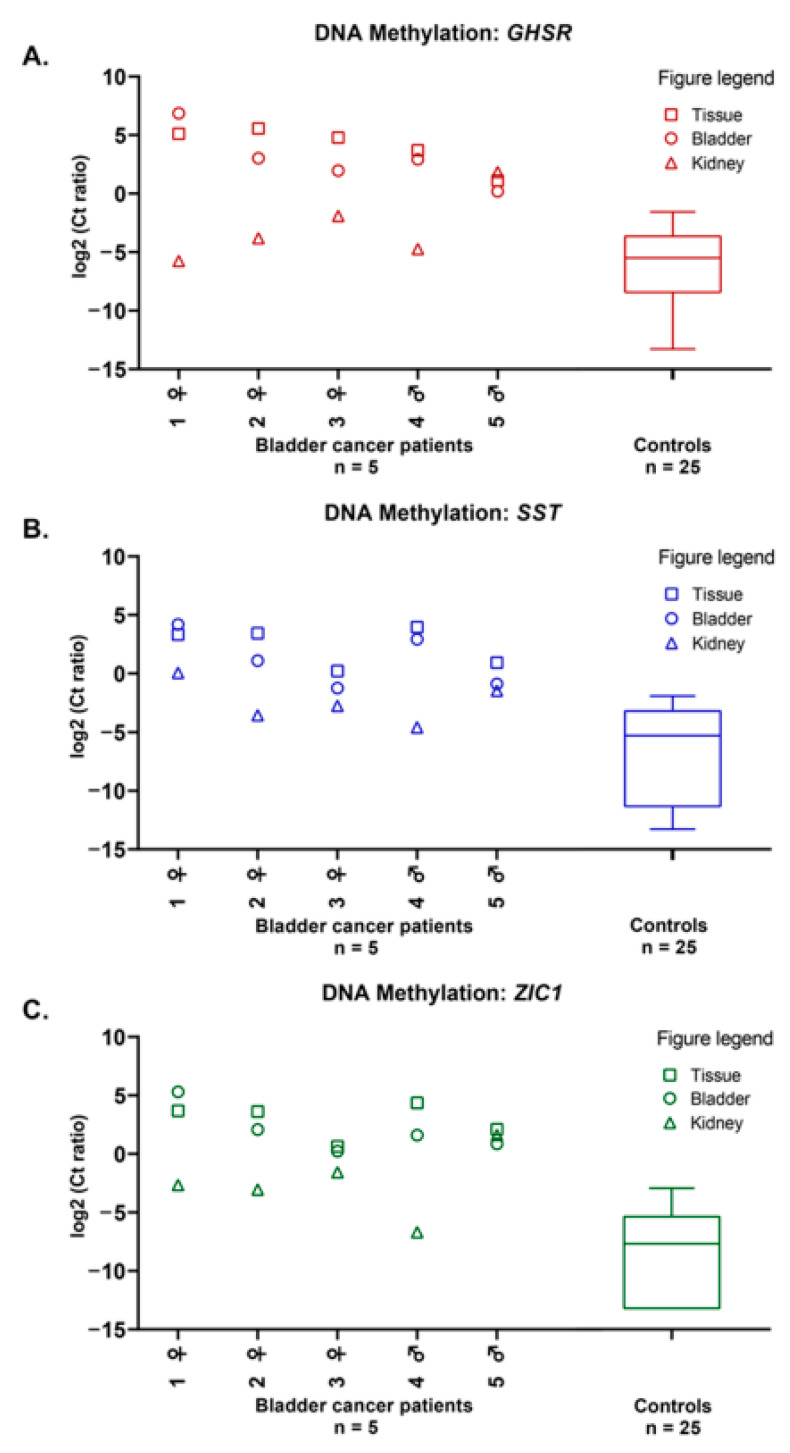
DNA methylation levels of markers *GHSR* (**A**), *SST* (**B**), and *ZIC1* (**C**) in paired bladder tumor tissues (tissue, **□**), natural voided urine (bladder, ○), and nephrostomy urine (kidney, ∆) from five muscle-invasive bladder cancer patients, compared to natural voided urine from 25 gender- and age-matched controls. DNA methylation levels are represented by the log2-transformed Ct ratios. Boxplots show medians with the 25th and 75th percentile and whiskers range from the smallest to the largest value.

Methylation levels of the markers *GHSR*, *SST,* and *ZIC1* were determined in bladder tumor tissues, in both paired natural voided urine and nephrostomy urine. Figure 1A–C shows that high methylation levels were found in all bladder tumor tissues and that methylation levels of the natural voided urine corresponded well to the respective bladder tumor tissues. Generally, the methylation levels of all three markers were higher in natural voided urine than in nephrostomy urine.

Next, methylation levels in the natural voided urine and nephrostomy urine of bladder cancer patients were compared to the natural voided urine of benign hematuria controls with similar gender (χ^2^-test: *p* = 0.854) and age (Mann–Whitney U test: *p* = 0.077) (Figure 1A–C, Table S1). We found that the methylation levels of markers *GHSR*, *SST,* and *ZIC1* were higher in natural voided urine (all, Mann–Whitney U test: *p* < 0.001) of bladder cancer patients than in the natural voided urine of benign hematuria controls. Furthermore, *SST* and *ZIC1* had significantly higher methylation levels in the nephrostomy urine (Mann–Whitney U test: *p* = 0.027 and *p* = 0.005, respectively) of bladder cancer patients than in the natural voided urine of benign hematuria controls, while for *GHSR* the difference was not significant (Mann–Whitney U test: *p* = 0.079). In bladder cancer patients 3 and 5, high methylation levels were found for all three markers in nephrostomy urine (Figure 1A–C).

### 2.2. Methylation Markers in Cervical Cancer

**Figure 2:**
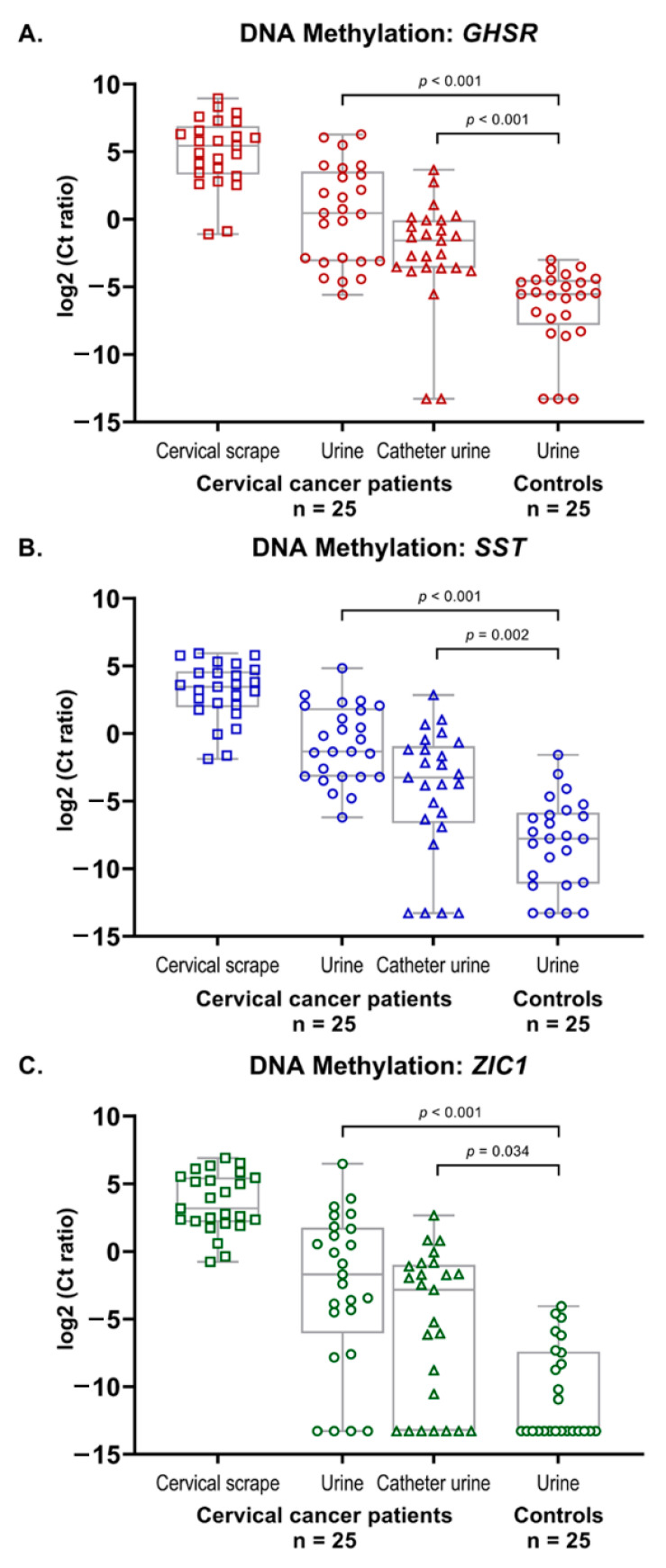
DNA methylation levels of markers *GHSR* (**A**), *SST* (**B**), and *ZIC1* (**C**) in paired cervical scrapes, natural voided urine, and catheter urine from 25 cervical cancer patients, as well as in natural voided urine from 25 healthy female controls. DNA methylation levels are represented by the log2-transformed Ct ratios. Boxplots show medians with the 25th and 75th percentile and whiskers range from the smallest to the largest value.

As shown in Figure 2A–C, methylation levels of markers *GHSR*, *SST,* and *ZIC1* were high in all cervical scrapes that were used as a reference. Methylation levels of markers *GHSR*, *SST,* and *ZIC1* were significantly higher in both natural voided urine (all, Mann–Whitney U test: *p* < 0.001) and catheter urine (Mann–Whitney U test: *p* < 0.001, *p* = 0.002, and *p* = 0.034, respectively) of cervical cancer patients as compared to the natural voided urine of age-matched controls. Furthermore, a significant increase was observed between the methylation levels of the paired samples in cervical cancer patients: from catheter urine, through natural voided urine, towards cervical scrapes (all, Friedman test: *p* < 0.001).

### 2.3. Mutation Markers in Bladder Cancer

**Figure 3:**
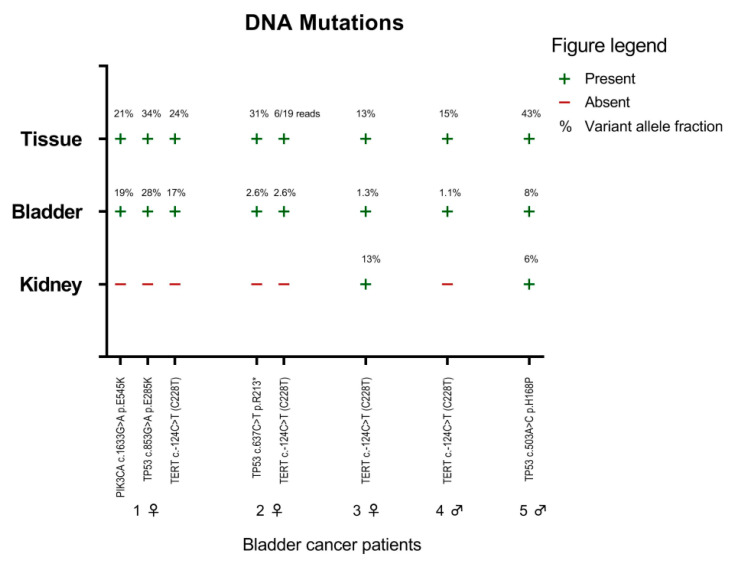
Presence of DNA mutations in paired bladder tumor tissues (tissue), natural voided urine (bladder), and nephrostomy urine (kidney) from five muscle-invasive bladder cancer patients. The percentage (%) indicates the variant allele fraction.

In each bladder tumor tissue, one to three mutations were detected in hotspot regions of three cancer-related genes: *PIK3CA*, *TERT* promoter, and *TP53* (Figure 3). The exact same mutations were also detected in the paired natural voided urine of all five bladder cancer patients. Moreover, in two bladder cancer patients, the same mutations were detected in nephrostomy urine. These were *TERT c.-124C>T (C228T)* in bladder cancer patient 3, and *TP53 c.503A>C p.H168P* in bladder cancer patient 5 (Figure 3).

## 3. Discussion

In this study, we showed that besides increased methylation levels in natural voided urine of both bladder and cervical cancer patients, increased methylation levels were also detected in paired nephrostomy and catheter urine. In both patient groups, the latter represents the transrenal urine fraction, as these collection methods excluded the local shedding of tumor DNA. Furthermore, DNA mutations present in bladder tumor tissues and natural voided urine in three genes known to be frequently mutated in bladder cancer [19,20] were also detectable in nephrostomy urine in a subset of patients. Hereby, we collected formal evidence that tumor DNA in the urine of patients with urogenital cancers is not only locally shed by tumor cells and cell fragments, but also excreted via glomerular filtration as cell-free tumor DNA.

Previous data showing the presence of Y-chromosome-specific sequences in the urine of women pregnant with a male fetus demonstrated that the blood–kidney barrier was permeable for circulating cell-free DNA [22]. Next to cell-free DNA, it was suggested that the permeability of the blood–kidney barrier also allowed for the transrenal passage of cell-free tumor DNA, as cell-free tumor DNA was identified in the urine of cancer patients other than urogenital cancer patients [23,24,25]. Although transrenal passage of cell-free tumor DNA in patients with urogenital cancers was predicted from our recent studies [6,7], we have now provided the first confirmation of this route.

The principle of local shedding is conventionally used in clinical practice for bladder and cervical cancer detection. In bladder cancer, the presence of tumor cells is investigated during the cytological examination of urine samples [26]. Although urine cytology is highly sensitive for the detection of high-grade tumors (84%), its sensitivity is insufficient for low-grade tumors (16%) [27]. In cervical cancer, exfoliated cells shed from the cervix are routinely collected by a cervical scrape and are currently used for diagnostics as well as for cervical cancer screening [28]. Tumor cells of gynecological origin have also been microscopically visualized in natural voided urine [29,30], which is most likely a consequence of shedding by exfoliated cells from the genital tract that are washed away from the vulva and urethra opening upon urination [5]. Present data demonstrating that the suitability of urine as a liquid biopsy for urogenital cancer detection relies on both the local shedding and transrenal excretion of tumor DNA paves an important way to non-invasive diagnostics in urogenital cancers. This is particularly significant since our findings are in accordance with results on other molecular biomarkers, which also showed the feasibility of urogenital cancer detection in urine sediment and urine supernatant [8,9].

One limitation of this study is the small number of bladder cancer cases, which is however inherent to the fact that the presence of a kidney nephrostomy is uncommon in bladder cancer patients, and therefore the sample collection of only five bladder cancer cases took over a year. For similar practical reasons, we had no access to nephrostomy and catheter urine of controls, hampering a valid comparison between cases and controls. For the evaluation of our hypothesis, we prioritized a homogeneous advanced population consisting of muscle-invasive bladder cancer patients. Although unlikely, results may not directly be translated to non-muscle-invasive bladder cancer patients. Furthermore, we cannot fully exclude the contamination of nephrostomy urine by vesicoureteral reflux in bladder cancer patients, or the contamination of catheter urine by positioning the transurethral catheter in cervical cancer patients.

A strength of this study is the collection of a unique set of paired samples of urogenital cancer patients. Moreover, we have included two cancer types with different anatomical positions in which local shedding of tumor DNA into the urine could be bypassed in order to further support our hypothesis. Lastly, methylation results were confirmed by mutation analysis in bladder cancer patients. Present findings warrant further studies into the mechanism of transrenal excretion of tumor DNA, which will be instrumental to the growing interest in non-invasive diagnostics in the field of oncology.

## 4. Materials and Methods

Bladder cancer patients and benign hematuria controls were included at the Department of Urology, Amsterdam UMC, location VUmc. Cervical cancer patients were included at the Department Gynecologic Oncology, Antoni van Leeuwenhoek/Netherlands Cancer Institute, Amsterdam. The healthy female controls were included in the Urine Control (URIC) biobank at the Department of Pathology, Amsterdam UMC, location VUmc. Prior to inclusion, written informed consent was obtained from all participants. The study was in accordance with the Declaration of Helsinki, and the research protocols were approved by the Medical Ethics Committee of Amsterdam UMC, location VUmc (bladder: no. 2018.355, controls: no. 2018.657) and the Medical Ethics Committee of Antoni van Leeuwenhoek/Netherlands Cancer Institute (no. METC15.1468/X15MET).

### 4.1. Sample Collection: Bladder Cancer

All five bladder patients had muscle-invasive disease and were TNM (2017) staged with T-stage at radical cystectomy ranging from pT2b to T4a, N-stage from pN0 to N2, and with no signs of (distant) metastasis present on computed tomography of thorax/abdomen. Two males and three females were included, with a median age of 73 years (interquartile range (IQR): 68–78 years) (Table S1). Natural voided urine and urine derived from the kidney nephrostomy were simultaneously collected prior to surgery. Bladder tumor tissues were collected during surgery as part of standard clinical practice. The median age of the benign hematuria control cohort was 63 years (IQR: 58–75 years).

### 4.2. Sample Collection: Cervical Cancer

All 25 cervical cancer patients were FIGO (The International Federation of Gynecology and Obstetrics, 2009) staged Ib1–IVb with a median age of 51 years (IQR: 42–68 years) (Table S1). The median age of the female healthy control cohort was 52 years (IQR: 41–59 years). Natural voided urine was self-collected by participants prior to surgery. During surgery, paired catheter urine and cervical scrapes were collected by the appointed physician. The presence of cancer was confirmed by histopathological reports after surgery.

### 4.3. Urine and Tissue Samples

Preservation of urine samples was achieved by the addition of ethylenediaminetetraacetic acid (final concentration 40 mM) [31]. For each patient, 15 mL urine was centrifuged at room temperature at 800× *g* for 10 min to obtain a urine supernatant and urine sediment, then both were stored at −20 °C. We used urine supernatant for bladder cancer patients and, for practical reasons, urine sediment for cervical cancer patients.

Tissue samples were fixed in formalin and embedded in paraffin in accordance with standard clinical practice. Archived whole tumor tissues were serially sectioned (10 μm) following the “sandwich cutting technique” [32]. The presence of tumor tissue was verified in the first and last obtained section (3 μm) after hematoxylin and eosin staining. For each patient, the remaining sections were collected in sterile polymerase chain reaction (PCR) tubes for DNA isolation.

Cervical scrapes were collected with a Cervex-Brush (Rovers Medical Devices, Oss, The Netherlands) in Thinprep PreservCyt solution (Hologic, Bedwork, MA, USA), and stored at 4 °C.

#### 4.3.1. DNA Isolation

DNA isolation from urine supernatant was performed with the QuickDNA™ Urine Kit (Zymo Research, Orange, CA, USA). DNA was isolated from urine sediment with the QIAamp DNA Mini Kit (Qiagen GmbH, Hilden, Germany), and from histological tissues with the QIAamp DNA FFPE Tissue Kit (Qiagen GmbH, Hilden, Germany). All samples were eluted in 50 μL elution buffer. DNA from cervical scrapes was isolated by the Microlab Star robotic system (Hamilton, Germany). All procedures were performed according to the recommendations of the manufacturer.

#### 4.3.2. DNA Methylation Analysis

DNA concentrations were measured using NanoDrop 1000 (ThermoFisher Scientific, Waltham, MA, USA) and DNA bisulphite conversion was performed with the EZ DNA Methylation™ Kit (Zymo Research, Orange, CA, USA), according to the manufacturer’s protocol. Multiplex quantitative methylation-specific PCR (qMSP) for the target genes *GHSR*, *SST,* and *ZIC1* was performed as described previously [16,18,33].

Both positive controls (bisulphite converted DNA of bladder cancer cell lines *TCC-SUP* and *J82*; bisulphite converted DNA of cervical cancer cell line *SiHa*), and a negative control (H_2_O) were included. Methylation values of the target genes *GHSR*, *SST,* and *ZIC1* were normalized to the reference gene *ACTB* with the comparative Ct method (2^−∆CT^ × 100) to calculate Ct ratios of the target genes. Two benign hematuria controls did not meet the sample quality criterion, defined as *ACTB* Ct < 32, and were therefore excluded from methylation analysis.

#### 4.3.3. DNA Mutation Analysis

Targeted next-generation sequencing (NGS) was performed on cell-free DNA isolated from urine supernatant and genomic DNA isolated from tissue samples of all bladder cancer patients. We used a customized sequencing panel (Ion AmpliSeq™ Custom panel, Life Technologies, Bleiswijk, The Netherlands) targeting mutation hotspot regions in 37 cancer-related genes (Table S2), including the 7 most important target genes for urinary bladder cancer diagnosis, in accordance with the results of our recent systematic review on this topic [20]. Barcoded DNA sequencing libraries were constructed using the Ion AmpliSeq™ Library Kit 2.0 and Ion Xpress™ Barcodes adapters kit (Life Technologies) according to the manufacturer’s protocol and subsequently purified using AMPure XP Reagent (Beckman Coulter, Indianapolis, IN, USA). The uniquely barcoded library samples were then amplified, purified, and equimolarly pooled for sequencing. The Ion Chef System (ThermoFisher Scientific, Landsmeer, The Netherlands) was used with the Ion PGM™ Hi-Q™ View Chef Kit (ThermoFisher Scientific) for fully automated template preparation and Ion 316 chip loading. Sequencing was performed using the Ion PGM Hi-Q Sequencing Kit on the Ion Torrent Personal Genome Machine System (ThermoFisher Scientific). Torrent suite software v5.12.1 was run for signal processing, quality reports, and to generate BAM files. Post-sequencing analysis was done with SeqNext software v4.2.1 (JSI Medical Systems GmbH, Ettenheim, Germany). Table S3 provides an overview of the average number of reads per sample type. For the 7 genes of interest, the average number of reads was 4335 (range 16–16,365) for bladder tumor tissue, 4798 (range 79–15,764) for natural voided urine, and 17,527 (range 607–83,820) for nephrostomy urine. A mutation was considered “present” at a minimum variant allele fraction of 1.0%. In principle, we handled a minimum number of 100 reads per amplicon for successful sequencing. If, however, the number of reads per amplicon was below 100 in one patient sample, but the mutation was called in the other two paired (urine/tissue) patient samples, the mutation was also considered “present” in the sample with insufficient reads.

#### 4.3.4. Statistical Analysis

Patient and tumor characteristics were described. The χ^2^-test was used to determine the difference in gender between bladder cancer patients and benign hematuria controls. Methylation levels were visualized and analyzed with log2-transformed Ct ratios. The non-parametric Mann–Whitney U test was performed to assess the differences in DNA methylation levels between cancers and controls. The Friedman test was used to analyze the differences in methylation levels between paired catheter urine, natural voided urine, and cervical scrapes collected from cervical cancer patients. *p*-Values < 0.05 were considered to be statistically significant. Statistical analyses were performed with SPSS Software (SPSS 26.0, IBM Corp., Armonk, NY, USA) and graphs were created with GraphPad Software (GraphPad Prism 8.2.1, San Diego, CA, USA).

## 5. Conclusions

We demonstrated that the feasibility of urine as a liquid biopsy for the non-invasive detection of urogenital cancers both relies on local shedding of tumor cells and cell fragments, and on transrenal excretion of tumor DNA. This underlines the great potential of urine as a liquid biopsy for urogenital cancer detection in future clinical practice.

## 6. Patents

Jakko A. Nieuwenhuijzen and Renske D.M. Steenbergen are inventors on patents related to the work.

## Data Availability

Data available on request.

## Supplementary Materials

The following are available online at https://www.mdpi.com/2072-6694/13/3/535/s1, Table S1: Age and gender of the included patients and controls; Table S2: Oncopanel V3 targets mutation hotspot regions in 37 cancer-related genes with 120 amplicons encompassing 13.46 kb target region. Oncopanel V3 includes the 7 most important target genes for urinary bladder cancer diagnosis, in accordance with the results of our recent systematic review on this topic [20]; Table S3: Overview of the average number of reads per sample type.
