# Supplementary material for: The Origin of Tumor DNA in Urine of Urogenital Cancer Patients: Local Shedding and Transrenal Excretion
Source: Cancers (Basel). 2021 Jan 31;13(3):535. doi: 10.3390/cancers13030535 (PMC7866784; doi:10.3390/cancers13030535)
Supplement: Supplementary file 1 [file cancers-13-00535-s001.zip › Steenbergen_table S3_revised.docx]

**Table S3**: Overview of the average number of reads per sample type.

| **Sample type** | **37 cancer-related genes** | | |
| --- | --- | --- | --- |
|  | **Average Read Coverage** | **Minimum** | **Maximum** |
| Bladder tumor tissue | 4904 | 16 | 20389 |
| Natural voided urine | 4628 | 2 | 15764 |
| Nephrostomy urine | 17016 | 16 | 91821 |
|  | **7 genes of interest** | | |
|  | **Average Read Coverage** | **Minimum** | **Maximum** |
| Bladder tumor tissue | 4335 | 16 | 16365 |
| Natural voided urine | 4798 | 79 | 15764 |
| Nephrostomy urine | 17527 | 607 | 83820 |
|  | **FGFR3** | | |
|  | **Average Read Coverage** | **Minimum** | **Maximum** |
| Bladder tumor tissue | 2234 | 701 | 4960 |
| Natural voided urine | 5983 | 1452 | 15764 |
| Nephrostomy urine | 19973 | 2808 | 77215 |
|  | **HRAS** | | |
|  | **Average Read Coverage** | **Minimum** | **Maximum** |
| Bladder tumor tissue | 1860 | 839 | 3006 |
| Natural voided urine | 5845 | 1842 | 12161 |
| Nephrostomy urine | 16669 | 3256 | 51580 |
|  | **KRAS** | | |
|  | **Average Read Coverage** | **Minimum** | **Maximum** |
| Bladder tumor tissue | 7493 | 1768 | 11893 |
| Natural voided urine | 3625 | 79 | 8083 |
| Nephrostomy urine | 15061 | 607 | 66358 |
|  | **NRAS** | | |
|  | **Average Read Coverage** | **Minimum** | **Maximum** |
| Bladder tumor tissue | 6115 | 1313 | 10253 |
| Natural voided urine | 3243 | 620 | 6160 |
| Nephrostomy urine | 12919 | 1174 | 44806 |
|  | **PIK3CA** | | |
|  | **Average Read Coverage** | **Minimum** | **Maximum** |
| Bladder tumor tissue | 8249 | 3703 | 16365 |
| Natural voided urine | 3961 | 592 | 11230 |
| Nephrostomy urine | 14388 | 1760 | 65879 |
|  | **TERT promoter** | | |
|  | **Average Read Coverage** | **Minimum** | **Maximum** |
| Bladder tumor tissue | 310 | 16 | 546 |
| Natural voided urine | 1268 | 315 | 2057 |
| Nephrostomy urine | 5878 | 1988 | 14918 |
|  | **TP53** | | |
|  | **Average Read Coverage** | **Minimum** | **Maximum** |
| Bladder tumor tissue | 2962 | 1229 | 5609 |
| Natural voided urine | 5678 | 1295 | 13317 |
| Nephrostomy urine | 21891 | 1690 | 83820 |
